# A scoping review on the use of different blood sources and components in the artificial membrane feeding system and its effects on blood-feeding and fecundity rate of *Aedes aegypti*

**DOI:** 10.1371/journal.pone.0295961

**Published:** 2024-01-22

**Authors:** Yuvaraahnee Suresh, Aishah Hani Azil, Syamsa Rizal Abdullah

**Affiliations:** Department of Parasitology and Medical Entomology, Faculty of Medicine, Universiti Kebangsaan Malaysia, Cheras, Kuala Lumpur, Malaysia; Al-Azhar University, EGYPT

## Abstract

In some laboratories, mosquitoes’ direct blood-feeding on live animals has been replaced with various membrane blood-feeding systems. The selection of blood meal sources used in membrane feeding is crucial in vector mass rearing as it influences the mosquitoes’ development and reproductive fitness. Therefore, this scoping review aimed to evaluate the existing literature on the use of different blood sources and components in artificial membrane feeding systems and their effects on blood-feeding and the fecundity rate of *Ae*. *aegypti*. A literature review search was conducted by using PubMed, Scopus, and Web of Science databases according to the Preferred Reporting Items for Systematic Reviews and Meta-Analyses (PRISMA-ScR). The EndNote version 20 software was used to import all searched articles. Relevant information was retrieved for analysis into a Microsoft Excel Spreadsheet. A total of 104 full-text articles were assessed for eligibility criteria, whereby the articles should include the comparison between different types of blood source by using the membrane feeding systems. Only 16 articles were finally included in the analysis. Several studies had reported that human blood was superior in blood-feeding *Ae*. *aegypti* as compared to sheep blood which resulted in lower fecundity due to accumulation of free fatty acids (FFA) in the cuticles. In contrast, cattle whole blood and pig whole blood showed no significant differences in the blood-feeding and fecundity rate as compared to human blood. This review also indicated that bovine whole blood and pig whole blood enhanced *Ae*. *aegypti*’s vitellogenesis and egg production as compared to plasma and blood cells. In addition, human blood of up to 10 days after the expiration date could still be used to establish *Ae*. *aegypti* colonies with good blood-feeding rates and number of eggs produced. Thus, future studies must consider the importance of selecting suitable blood sources and components for membrane blood feeding especially in mosquito colonisation and control measure studies.

## Introduction

*Aedes aegypti*, the primary arbovirus vector which carries diseases such as dengue, Zika, and chikungunya, has a wide geographic range and can be found in tropical and subtropical areas all over the world [1, 2]. Even though they are most prevalent in humid tropics and subtropics, mosquitoes also threaten public health in temperate regions [3, 4]. The changes in vector presence and abundance are influenced by weather factors and climate change [5]. This leads the public and scientific community to become more concerned about mosquito-borne diseases due to their rapid geographic distribution and rising disease burden. In 2022, the World Health Organisation (WHO) estimated that 390 million people were infected with the dengue virus annually, with Asia accounting for 70% of those cases. The most concerning aspect is that the number of cases continues to increase annually, with 2019 having the highest number of dengue cases recorded worldwide [6].

Effective vector management techniques and extensive surveillance are essential for attaining and maintaining a decline in dengue-related morbidity as the preventive and vector control strategies attempt to minimise dengue transmission, lower the frequency of illness, and prevent disease outbreaks [7]. Therefore, studies on mosquito-borne diseases play an important role in providing effective, efficient and environmentally proper mosquito control [8]. As a result, a large number of insectary-reared mosquitoes is needed for research, mosquito control programmes and studies which involve genetically modified and disease-resistant mosquitoes. Consequently, blood-feeding is a crucial method to produce insectary-reared mosquitoes, whereby an anautogenous mosquito like *Ae*. *aegypti* must consume at least one blood meal from a vertebrate host to successfully produce and lay one clutch of eggs [9].

The blood-feeding of female mosquitoes is an essential part of colonisation as it allows female oocytes to develop and mature [10, 11]. The blood-feeding process consists of four sequential steps, including being attracted to and settling on the host, piercing the host’s skin with a fascicle, sucking blood, and removing the styles [12]. Therefore, the blood-feeding process is one of the most complicated and difficult steps to imitate when rearing mosquitoes in the laboratories.

Traditional methods, such as by using the forearm of an entomologist or willing volunteer, may present risks, for example, if the mosquito colony or person feeding them is unknowingly infected with a pathogen [13, 14]. Besides that, various species of live animals also used for mosquito blood feeding in the laboratory, including birds, mice, hens, rats, guinea pigs, white rabbits, hamsters, sheep, horses, and even humans [15–22]. However, by using anaesthetised live animals for direct blood-feeding in the laboratory has several drawbacks, such as the pain and stress caused by blood-feeding, the high cost of keeping animals in the laboratory, need for a suitable physical environment for animals, and specialised staff to manage these resources [10, 23]. In practice, these approaches also frequently require animal preparation, like removing or shaving off its hair, restraints, sedatives and anesthetics, hence, making the process more complicated [24]. In addition, when it comes to animal usage, bioethics committees urge the application of 3Rs: replacement, reduction and refinement. Also, they suggest the use of artificial blood-feeding systems instead of live animals [25].

As a result, several artificial membrane blood-feeding systems were developed to maintain *Ae*. *aegypti* colonies in the laboratory. These ranged from commercial blood feeding devices, such as the Hemotek membrane feeding system [26] that can cost up to £980 to £2510 [27] to more cost-effective techniques [28–31]. Besides being low in cost, this artificial blood-feeding system often uses a membrane to mimic the host skin and is effective for blood-feeding without the requirement for maintaining live hosts. Therefore, to induce mosquitoes to feed on blood by using an artificial blood-feeding system require a few components, such as blood source, membrane, feeder which can hold the blood and heat source [11]. When rearing mosquito vectors in the laboratories, researchers frequently face two challenges, which are the feeding technique and blood source that will significantly provide consistency and success in colony establishment, as well as experimentation [32]. Several studies had demonstrated effective artificial methods for blood-feeding *Ae*. *aegypti* in the laboratory settings. However, there is currently a notable gap in research concerning the preferred blood sources of *Ae*. *aegypti* and how these sources impact its blood-feeding and fecundity rate.

Therefore, the purpose of this review is to evaluate the data (from peer-reviewed papers) on the use of different blood sources and components in artificial membrane feeding system and their effects on blood-feeding and fecundity rate of *Ae*. *aegypti*. These study findings may help various research institutes to look for suitable blood sources that will provide the best growth and reproduction rates for *Ae*. *aegypti* species.

## Methods

The methodologies used in this scoping review were developed by Joanna Briggs Institute (JBI), and are based on a framework first proposed by Arksey and O’Malley (2005) and refined by Levac et al. (2010) [33–35] According to the JBI structure, six steps should be included in a scoping study to assist authors: (i) formulating a research question, (ii) finding relevant studies, (iii) study selection, (iv) visualising data, (v) combining, summarizing, reporting results and (vi) validating study findings [35]. The following research question was investigated by using population, concept, and context (PCC): In artificial membrane feeding of *Ae*. *aegypti* (population), do different types of blood source and component used (concept) affect the blood-feeding and fecundity rate of this species (context)? The PCC framework was selected because it facilitated more detailed and accurate findings. The included studies complied with the following criteria:

### Population

Only *Aedes aegypti* species that had been blood fed by using an artificial membrane feeding system were included irrespective of any countries. Each article was screened to determine if the population met the eligibility requirements. Articles that included other mosquito species with artificial membrane feeding were excluded from this review.

### Concept

This scoping review focused on the types of blood source and component used in artificial blood-feeding system to blood feed *Ae*. *aegypti*. Articles with direct blood-feeding on different types of live animals were excluded.

### Context

The reviewers included publications that had reported the effect of the concept on (i) blood- feeding rate [(total number of engorged female mosquitoes / total number of female mosquitoes) x 100] and (ii) fecundity rate (number of eggs laid by female mosquitoes)].

### Search strategy

This scoping review did not focus on the method of blood-feeding *Aedes* mosquitoes, but on studies that contained information on types of blood source and component used in the artificial membrane feeding system. The PubMed, Scopus, and Web of Science (Clarivate Analytics) databases were searched for articles from January 1980 to January 2023. In order to make the review more comprehensive and reliable, backward citation screening was used to find eligible articles from each database and then manually searched for more articles. The keywords were combined with suitable Boolean operators like “AND” and “OR” to search for articles in the electronic databases. In order to make the search more precise, proximity operators, truncation, and wildcards were applied to the keywords for the final search strings.

### Eligibility criteria

Only full-text articles in English were taken into consideration in this scoping review, whereby articles from countries that were published in a different language might have been overlooked. Besides that, systematic, scoping or narrative reviews and meta-analyses were excluded as the study only interested in the findings of a single study. In the full-text screening stage, reference lists of all of these “reviews” were looked through to find more primary research on the study topic. Moreover, the searched articles must describe about the mosquito species, study sample size, study design, blood-feeding methodology and parameters measured.

For exclusion criteria, an article was excluded if (i) it was not available in full-text, (ii) it contained only mosquito blood-feeding techniques or methods without reporting any results of different types of blood source or component, and (iii) its intended parameters like blood-feeding rate and egg production were not reported. In addition, literature reviews and articles with inadequate data were also excluded.

### Data extraction and data charting

The EndNote version 20 software was used to import all searched articles while duplicate files were deleted. All articles and other data files were safely stored in Google Drive by the reviewer. The studies were chosen independently by three reviewers and any discrepancies were handled in discussions with the team. The articles were evaluated twice, whereby the first screening step was based on the title and abstract of article, followed by the full-text screening step. Then, relevant information was retrieved for analysis by using the Microsoft Excel Spreadsheet. The data extraction sheet included the first author’s name and publication year, title of article, types of blood-feeding used, study design, types of blood source or blood component used, and the key findings.

## Results

### Description of studies

**Fig 1:**
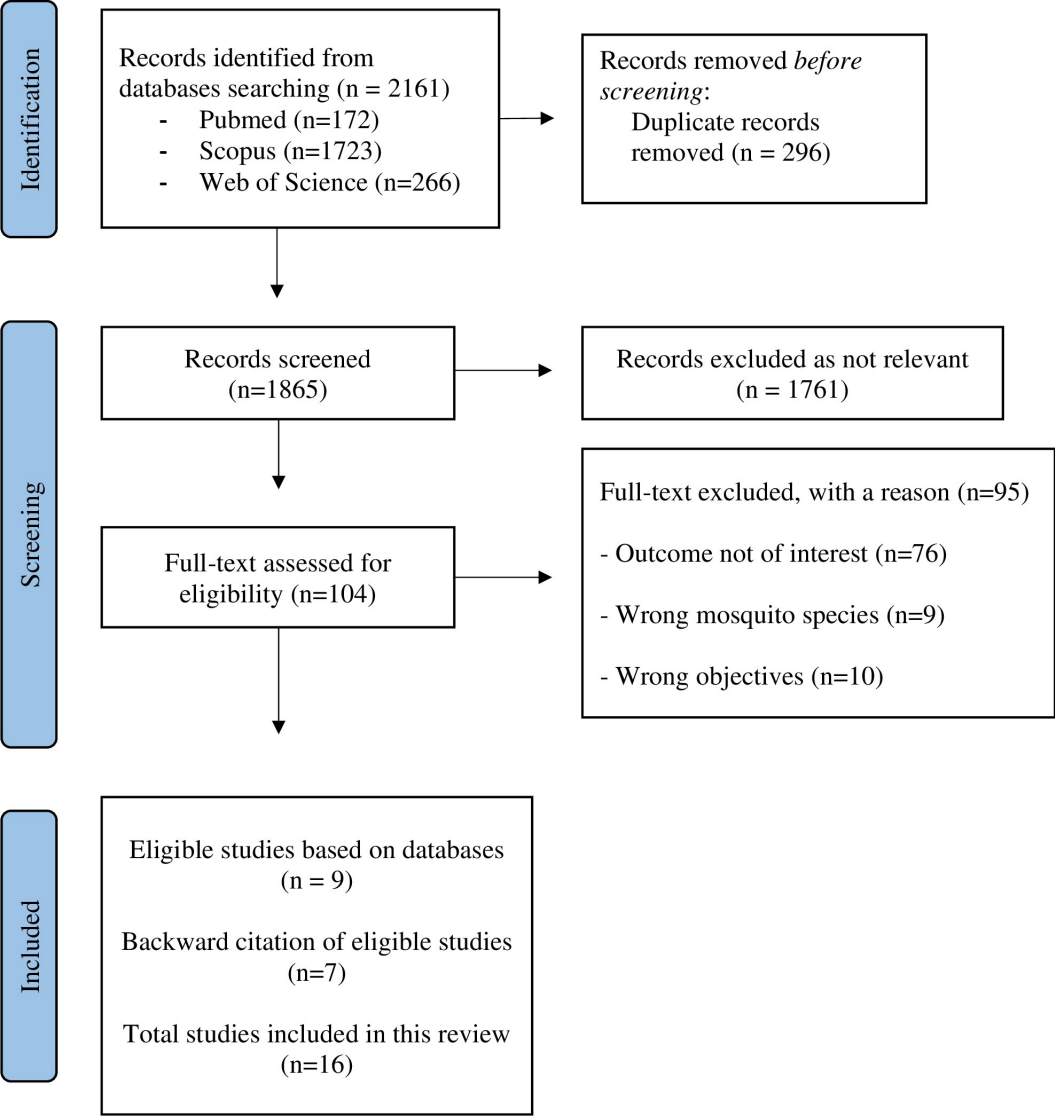
PRISMA 2020 flow diagram.

In the initial search through the three databases, a total of 2,161 articles were identified by the reviewers. After the duplicates (n = 296) were removed, the remaining articles were screened by reading the abstracts and publication titles. Then, the remaining 104 full-text articles were further screened for eligibility. Following the final full-text screening, nine articles were selected for inclusion and seven more articles were included following backward citations from these nine articles, resulting in total of 16 eligible articles. Fig 1 shows a flow diagram of the selection process. The most common reasons for exclusion were that the outcome was not of interest and different mosquito species. Some other exclusion criteria were unrelated objectives, reviews, and experimental studies.

The PRISMA flow diagram shows the search and selection process used during our systematic literature search for this scoping review.

### Characteristics of included studies

In the selected studies, different types of artificial blood-feeding technique were used: two used Hemotek membrane feeding system [36, 37], two used Glytube [38, 39], two used metal plate [11, 26] and 10 other studies used a variety of simple and affordable membrane feeding methods, which consisted of different types of membrane, such as the sausage casing membrane [12, 20, 30], and Parafilm M [10, 40, 41]. Besides that, several heating sources for the blood, namely circulating water bath [10, 20, 42, 43], germination mat [11], rice grains [26], vegetable oil bag [40], USB chargeable hand warmer, [44] and microwavable heat pack [45] were also used.

A total of 11 studies from 16 used direct blood-feeding method on live animals, such as mice [12, 36, 38, 39, 41, 44], guinea pigs [10, 30], hamsters [20, 42] and rat [40] as the study control. Furthermore, many blood sources from different vertebrates like guinea pigs, mice, rat, chicken, turkey, pig, sheep, cattle, humans as well as blood components such as whole blood, plasma, blood cells from chicken, turkey, rat, pig, sheep, cow and humans were used in the artificial membrane blood feeding system. Besides that, there were a variety of parameters measured in the selected studies such as blood-feeding rate, fecundity rate, hatching rate, survival rate, oviposition rate, mean time egg to adult, the ratio of indigested blood, pupal emergence rate and yolk deposition. However, only blood-feeding and fecundity rate were included in this review because they were reported in all studies. Table 1 summarises the types of study that were taken into account for the review.

**Table 1:** Summary of included studies.

Ref.	Author	Types of membrane blood feeding	Control	Types of blood sources used	Parameters measured
[20]	Phasomkusolsil et al., (2013)	Feeding cups with sausage casing membrane and circulating water bath	Direct blood feeding on hamster	Guinea pigs, human, and sheep blood	Blood feeding rate, fecundity, hatching, survival rate, and mean time egg to adult
[26]	Gunathilaka et al., (2017)	Metal plate	Human blood (from volunteer)	Cattle and chicken blood	Blood feeding rate, fecundity, and hatching rate
[10]	Dias et al., (2018)	Jacketed glass cones with Parafilm-M and circulating water bath	Direct blood feeding on guinea pigs	Defibrinated rabbit and sheep blood, citrated rabbit and sheep blood	Blood feeding rate, fecundity, hatching rate and the ratio of ingested blood
[36]	Farnesi et al., (2021)	Hemotek system	Direct blood feeding on mice	Mouse, guinea pig and human blood	Fecundity, and hatching rate
[39]	Costa et al., (2020)	Glytube	Direct blood feeding on mice	Chicken blood using different membranes	Blood feeding and fecundity rate
[37]	Paris et al., (2018)	Hemotek system	Human blood	Sheep blood and pig blood	Fecundity rate and hatching rate
[11]	Tyler-Julian et al., (2021)	Metal plate with Parafilm-M and PTFE tape with germination mat as heating device	-	Bovine blood and pork blood	Blood feeding rate, fecundity, and hatching rate
[38]	Costa et al., (2013)	Glytube	Direct blood feeding on mice	Human blood	Blood feeding rate
[30]	Deng et al., (2012)	Cattle collagen membrane casing with in-house designed heating device	Direct blood feeding on guinea pig	Mini pig blood	Blood feeding rate, fecundity, hatching, and survival rate
[40]	Finlayson et al., (2015)	Glass petri dish with parafilm M and pre-heated vegetable oil bag as heating device	Direct blood feeding on rat	Heparinized cow blood	Blood-feeding rate, oviposition and fecundity rate
[41]	Sri-In et al., (2020)	Parafilm blood feeder	Direct blood feeding on mice	Washed sheep blood, fetal bovine serum and adenosine triphosphate	Blood-feeding rate and fecundity rate
[42]	Pothikasikorn et al., (2010)	Jacketed glass cones with Parafilm-M and circulating water bath	Direct blood feeding on hamster	Out-of-date human blood	Oviposition rate, hatching rate, and pupation rate to adult formation rate
[12]	Luo (2014)	Multiple membrane blood feeding system	Direct blood feeding on mice	Pig whole blood and plasma (w/o ATP), pig whole blood and plasma (w/ ATP)	Blood-feeding rate, survival rate, fecundity rate, hatching rate, pupation, and pupal emergence rate
[44]	Harrison et al., (2021)	Caps of microfuge tubes with parafilm M and USB chargeable hand warmer as heating device	Direct blood feeding on mice	Whole blood, plasma, blood cells of human, rat, chicken, turkey and blood cells of cow, sheep, rat	Blood-feeding rate, fecundity rate and hatching rate
[45]	Gonzales et al., (2015)	Hemotek system	Defibrinated bovine whole blood	Bovine serum, red blood cell, hemoglobin, bovine serum albumin (BSA)	Blood-feeding rate and fecundity rate
[43]	Harrington et al., (2001)	Jacketed glass feeder with circulating water bath	Human forearm	High and low isoleucine human and chick blood, human and mouse plasma	Blood-feeding rate and fecundity rate

### Blood sources

Results from all included studies showed that blood meal sources affected blood-feeding and fecundity rate of *Ae*. *aegypti*.

#### Human blood

The mean percentage of blood feeding rate from feeding on human blood was, on average, high (mean ± standard deviation) (between 51.3% and 97.33 ± 0.99%) [20, 26, 38] which also resulted in a higher fecundity rate that ranged between 14.85 ± 0.39 and 101.0 ± 21.0 eggs per female mosquito, as compared to other blood meal sources.

#### Cattle blood

Cattle blood demonstrated a comparable blood-feeding rate which ranged between38.2 ± 21.5% and 90.12 ± 3.10% and egg production between 86.7 ± 7.8 and 162.32 ± 25.94 eggs per female with human blood [11, 26, 40].

#### Sheep blood

Even though sheep blood showed a higher blood feeding rate which ranged between 86.7 ± 8.5% to 97%, it negatively affected the number of eggs laid that resulted in a significantly low fecundity rate [10, 20]. Despite of low fecundity rate, sheep blood showed no significant difference in the hatching rate as compared to human blood and direct blood-feeding on hamster [20].

#### Chicken blood

Chicken blood showed a slight difference in both blood-feeding and fecundity rate as compared to human blood and direct blood-feeding on mice [26, 39]. Additionally, chicken blood showed a blood-feeding rate of 79.67 ± 0.91% using metal plate with Parafilm-M method [26] while the blood feeding rate was reduced to 45.00 ± 1.35% when using Glytube with Parafilm-M method [39]. Meanwhile, only 12.69 ± 0.28 eggs per female was produced in metal plate method whereas female mosquitoes blood fed using Glytube method produced 66.05 ± 15.11 eggs regardless of their low blood feeding rate.

#### Guinea pig blood

According to the findings by Phasomkusolsil et al. (2013), guinea pig blood with 66.7 ± 16.6% of blood-feeding rate showed no significant differences with human blood 67.0 ± 19.9% and it demonstrated a higher fecundity rate as compared to mouse blood and human blood in the study by Farnesi et al., [36].

#### Pig blood

Two studies found that the blood-feeding and fecundity rate of pig blood showed no significant differences with the control human blood [37] and direct blood-feeding on mice [12]. Similar findings were also found by Deng et al., (2012) when mini pig blood was used against direct feeding on guinea pigs as control.

#### Rabbit blood

Rabbit blood was used in only one study by Dias et al. (2018) [10] whereby the defibrinated and citrated rabbit blood showed no significant differences in blood-feeding and fecundity rate. However, it had a low hatching rate which ranged from 54% to 69%, as compared to the direct blood-feeding on guinea pig.

#### Expired human blood

A study by Pothikasikorn et al. (2010) [42] found a small significant difference in the blood-feeding and fecundity rate of *Ae*. *aegypti* blood that was fed on expired human blood of between 5 days and 15 days, as compared to the blood-fed mosquitoes on mice. However, the 25-day expired human blood showed a very low blood-feeding rate of 18% and 8.8 eggs per female mosquito.

### Blood components

In this review, three studies evaluated the efficiency of blood components in blood-feeding *Ae*. *aegypti* by using a membrane blood-feeding system [12, 44, 45].

#### Whole blood

According to the findings by Luo (2014) [12], the blood-feeding rate of pig whole blood by using Parafilm-M membrane (92.9%) and cattle collagen sausage-casing membrane (89.7%), pig whole blood added adenosine triphosphate (ATP) (90.7%) and pig plasma added with ATP (91.5%) showed a comparable rate with the control direct blood-feeding on mice (98.0%). Besides blood-feeding rate, the fecundity of per female fed on pig whole blood (49.88 ± 7.92 eggs) shows no significant differences with the fecundity rate fed on mice (58.33 ± 2.56 eggs) but it was higher than those fed on pig plasma (33.88 ± 6.43 eggs). In addition, pig whole blood demonstrated a higher hatching rate (94.10 ± 3.54%) than direct blood-feeding on mice (86.25 ± 4.60%) and pig plasma (77.00 ± 6.51%).

Apart from that, blood components like whole blood, plasma and blood cells from two mammals (humans, rat) and two birds (chicken, turkey) were evaluated for the efficiency in blood feeding *Ae*. *aegypti* [44]. Amongst the three blood components, whole blood of all vertebrates was identified as the most preferred blood components by *Ae*. *aegypti* in terms of blood-feeding and fecundity rate. Another study by Gonzales et al. (2015) [45], also found the similar findings, whereby bovine whole blood recorded the highest rate in blood-feeding and fecundity followed by bovine serum albumin and bovine serum [45].

#### Red blood cells, hemoglobin and plasma

Bovine red blood cells, bovine hemoglobin (Hb), sheep blood cells, chicken and turkey plasma recorded the lowest blood-feeding rate with no eggs produced. On the other hand, chicken and turkey blood cells stimulate more eggs production in *Ae*. *aegypti*, whereas human blood cells caused females to lay a few or no eggs [44].

#### Isoleucine content in blood source

There was no significant difference in the number of eggs produced per mosquito between females that were artificially fed on low isoleucine human blood (55.8 ± 10.6 eggs) as compared to the high isoleucine human blood (52.0 ± 4.4 eggs). It was similar for females that were fed on human naturally (low isoleucine) with a mean of 84.1 ± 5.3 eggs per female as compared to a chick (high isoleucine) with 83.6 ± 5.4 eggs [43].

## Discussion

Several research studies demonstrated that many factors influenced the physiological processes in mosquitoes, such as the species of mosquito [46], body size of species [47], blood meal size [48], amino acids derived from erythrocytes [49], types of artificial blood-feeding systems and synthetic membranes [26, 39], types of blood sources or components [44], and the anticoagulant used to preserve the blood source [50]. It had been discovered that mosquito meals must contain proteins for egg maturation and oogenesis [10, 51–53].

*Ae*. *aegypti* prefers mammalian blood [54] selectively human blood [26], even under the availability of other blood sources [55]. However, a previous study which used blood-feeding *Ae*. *aegypti* revealed that avian blood resulted in higher egg production than mammalian blood [56]. Contrarily, Suleman and Shirin (1981) [57] found that mammalian blood increased fertility more than avian blood in *Culex quinquefasciatus*. Therefore, from all studies included in this review, human whole blood-fed mosquitoes showed higher blood-feeding, fecundity and hatching rates as compared to other blood sources or components. However, there are a number of ethical and safety concerns with the use of human blood for insectary colony management. Human blood is also in short supply due to lack of donors, whereby human blood preserved in blood banks is primarily used for medical emergencies instead of insectary experiments. Apart from that, the use of live animals such as mice [12, 36, 38, 39, 41, 44], hamsters [20, 42], guinea pigs [10, 30], and rats [40] in direct blood feeding of *Ae*. *aegypti* also gives good blood-feeding and fecundity rates. However, the use of animals in these insectary experiments poses several challenges, including in maintaining the resources, which is costly and labour-intensive, and thus requires approval from animal ethics committee [10]. Therefore, various membrane blood feeding systems were developed, and suitable blood sources or components are required as a blood meal source.

Several studies were conducted to investigate the variation in fertility of mosquitoes after feeding on various hosts, as reviewed by Lyimo and Ferguson (2009) [58]. Subsequent research revealed that this variation was caused by different levels of amino acids in different vertebrate blood sources, whereby some amino acids are limiting factors for egg formation while others enhance the vitellogenesis in mosquitoes [59, 60]. A study by Kaczmarek et al. (2021) [50] found that artificially sheep blood-fed mosquitoes showed significantly higher levels of free fatty acid content in the cuticle of *Ae*. *aegypti* as compared to human blood-fed mosquitoes [50]. As a result, the artificially sheep blood-fed mosquitoes showed a reduction in fecundity rate, which might be related to increased transport and buildup of free fatty acids in the cuticle. So, these modifications in the mosquitoes’ body might eventually disrupt the fertility and sensitivity of these insects towards chemical substances like insecticide.

Besides that, a study by Harrington et al. (2001) [43] showed that low isoleucine human blood might increase the energy stores as well as the physiological processes in *Ae*. *aegypti* instead of high isoleucine rodent blood or avian blood. However, the differences in energy stores were less noticeable when the females were artificially given human blood with or without isoleucine added. This indicated that a low isoleucine level in human blood might not be the only component which helps energy reserves build up [43].

Bovine whole blood and pig whole blood showed no significant difference in blood-feeding rate as compared to the controls [12, 37, 45]. It was also found that *Ae*. *aegypti* fed on bovine whole blood, serum and bovine serum albumin (BSA) produced a greater number of eggs per female as compared to those fed on red blood cells (RBCs) or hemoglobin (Hb), which resulted in no eggs. In contrast, Luo (2014) [12] found that *Ae*. *aegypti*’s fecundity rate was considerably reduced when fed with pig plasma as compared to pig whole blood. This was because if sedimentation took place in whole blood during blood-feeding of *Ae*. *aegypti* on artificial feeding system, it might result in fewer eggs as they might consume plasma rather than whole blood [12]. Therefore, bovine whole blood and pig whole blood can routinely be obtained from a nearby slaughterhouse and stored in the refrigerator for a period of time, depending on the anticoagulant used [26, 61]. However, this strategy is not practical for certain institutions that are located far from the slaughterhouse.

Apart from that, frozen citrated bovine blood that can be purchased from grocery stores was also successfully used to establish *Ae*. *aegypti* colonies with a higher fecundity rate, whereby the frozen bovine blood was thawed before use [11]. This frozen bovine blood can be stored for up to one year in the freezer, which will be a good option for research institutes which are located far from the slaughterhouse [11]. In addition, expired human blood can be obtained from blood banks or hospitals, whereby it has been successfully utilised in the blood-feeding of *Ae*. *aegypti* [23, 42, 62]. It is proposed that, *Ae*. *aegypti* may be fed on human blood that has been stored for up to 10 days past its expiration date [42]. Furthermore, the previous study revealed that fresh blood had no advantages over preserved human blood as long as the red blood cell lysis does not occur during collection and preservation.

In addition, the types of artificial blood-feeding system have been proven to influence the blood feeding rate and egg production of *Ae*. *aegypti* as the use of chicken blood in a metal plate with Parafilm-M resulted in a higher blood-feeding rate and lower egg production than Glytube with Parafilm-M [26, 39]. Likewise, the selection of synthetic membranes used in artificial blood-feeding systems also plays a significant role in mosquito blood feeding, whereby citrated bovine blood that used a metal plate with polytetrafluoroethylene (PTFE) showed a higher blood feeding and fecundity rate than metal plate with Parafilm-M [11]. Therefore, a further detailed review is needed to include all other factors that could affect the blood feeding and fecundity rate in membrane-feeding *Ae*. *aegypti*.

Consequently, from this scoping review, it is concluded that bovine whole blood and pig whole blood could be suggested as a suitable blood source in membrane feeding, followed by guinea pig blood in mosquito colonisation. However, other factors such as types of artificial feeding system, heat sources, synthetic membranes, duration of blood feeding, as well as the anticoagulant used play a vital role in selecting a suitable blood meal source. It is hoped that this scoping review provides a framework for future research that is looking for suitable blood meal sources or components to be used in artificial blood feeding systems.

### Limitations

This scoping review lies in the restriction to a limited number of databases, namely PubMed, Scopus, and Web of Science, for the identification and retrieval of relevant literature. This selective approach may have resulted in the inadvertent exclusion of valuable studies from other databases that could have contributed diverse viewpoints and a more comprehensive understanding of the blood sources preferred by *Ae*. *aegypti*. Additionally, only two parameters, namely blood-feeding and fecundity rate were considered within the scope of the included studies. This was because only these two common parameters were evaluated amongst all the 16 included studies. By limiting the assessment to these specific parameters there exists the possibility of neglecting other equally relevant parameters that are influenced by the blood sources and blood components fed by *Ae*. *aegypti*.

## Conclusions

This scoping review summarised the effects of different types of blood source and component on *Ae*. *aegypti*. It explores the impact on the blood-feeding and fecundity rates of *Ae*. *aegypti*. In this review, 16 eligible studies found that the blood-feeding and fecundity rates of *Ae*. *aegypti* were influenced by the types of blood source or component taken by the species. Therefore, bovine whole blood and pig whole blood showed a comparable blood-feeding and fecundity rate with human blood, followed by guinea pig’s blood. Besides that, the findings of this review showed that sheep blood was not suitable for use in membrane feeding as it decreased the fecundity rate of *Ae*. *aegypti*. The findings of this review will be useful to find suitable blood sources and components in blood-feeding *Ae*. *aegypti* which will certainly provide the best growth and reproduction rates that play a significant role in establishing mosquito colonies in studies related to medical entomology.

## Supporting information

S1 Table Listed initial papers assessed for eligibility. (XLSX)

S1 Checklist Preferred Reporting Items for Systematic reviews and Meta-Analyses extension for Scoping Reviews (PRISMA-ScR) checklist. (DOCX)
